# Monitoring of Post-Harvest Maturation Processes inside Stored Fruit Using Photoacoustic Gas Sensing Spectroscopy

**DOI:** 10.3390/ma13122694

**Published:** 2020-06-12

**Authors:** Ana Maria Bratu, Mioara Petrus, Cristina Popa

**Affiliations:** Laser Department, National Institute for Laser, Plasma and Radiation Physics, 409 Atomistilor St., PO Box MG-36, 077125 Magurele, Romania; mioara.petrus@inflpr.ro

**Keywords:** gas detection, gas sensing techniques, volatile organic compounds, infrared photoacoustic spectroscopy, COMSOL Multiphysics

## Abstract

Gases produced inside harvested fruit sensitively influence the continuing quality of the stored fruit and its maximum time of storability. In this work, the evolution of gaseous volatiles inside “Golden Delicious” apples were studied using CO_2_ laser photoacoustic spectroscopy with the aim of developing new methods for in-storage fruit quality monitoring. Studying the concentrations of volatile organic compounds generated inside “Golden Delicious” apples during storage, it was found that the concentrations of these compounds depended on the stage of maturity reached during fruit maturation and on the conditions of preservation. Numerical simulations using COMSOL Multiphysics software were used to study the conversion of ethylene to ethanol in the course of respiration processes occurring inside stored food. Experimental data obtained by means of photoacoustic spectroscopy were used to critically assess the simulation results. Using the combination of both techniques, new prospects for the development and implementation of advanced schemes of fruit storage and preservation have emerged.

## 1. Introduction

Fruits are well recognized for their importance to human health as these are good sources of energy, vitamins and minerals without which the human body cannot maintain proper health and resilience to disease [1]. Proper fruit storage helps to reduce the chances of suffering food-borne illnesses. To ensure optimal storage conditions for long periods of time it is necessary to provide adequate external environments and to investigate those biochemical processes that go on within the stored fruit themselves [2]. External environmental factors that can affect fruit storage are light and temperature while the internal factors are gases and volatiles diffusing through fruit tissue and controlling its maturation. The internal atmosphere of fruit has importance to postharvest physiology and food science and technology. The involvement of gases like ethylene and ethanol in fruit development has a crucial effect on the fruit’s maturation and storability [3]. The correct timing of the harvest, the maturity and the aging of the apples, for instance, may be best determined by monitoring the biosynthesis of fruit ethylene.

During development and maturation, fruit exhibit two distinct respiratory patterns and which allow fruit to be classified into climacteric and non-climacteric groups [4,5,6].

Climacteric fruit are those that can ripen after being picked and which show a dramatic increase in the respiration rate during ripening. In contrast, non-climacteric fruit are characterized by the impossibility of ripening once they have been removed from the plant [7,8].

Apple is a climacteric fruit and most of the relevant physiological changes are triggered and governed by the action of ethylene. To prolong the storage period, the maturation must be delayed by sorting, but also by storing the fruit at low temperatures and in controlled atmospheres. Under proper conditions and harvested at optimum storage potential, apples may be stored as long as 12 months [9].

The volatiles present inside the fruit are ethylene, ethanol, acetaldehyde, methanol, acetone, butanol, propanol, ethane, hexanol, sulfur compounds, ammonia, etc. These volatiles can be used to determine the optimum maturity stage for the harvesting of climacteric fruit. It is important to evaluate the significance of volatiles and their interaction with ethylene in postharvest situations. Different factors and conditions can influence the accumulation and release of these volatiles [10]. The volatile plant hormone ethylene is a hydrocarbon gas without scent and invisible to the eye. In fruit, ethylene is a gas emitted naturally in the process of ripening or produced upon inflicting plant injury. This potent molecule regulates the plant’s growth and development as well as the speed of this processes [11,12,13,14,15] at concentrations as low as about 0.01 to 1.0 parts per million (ppm). Climacteric products, such as tomatoes and apples, can produce tens of ppm of ethylene. Recent research aiming at improving the postharvest fruit quality, therefore, concentrates on the possibility of controlling the effect of ethylene [16,17].

Ethanol is a simple alcohol resulting from the fermentation of fruit sugars by yeast and occurs when O_2_ concentrations are very low [18]. The maturation of the fruit causes changes in the levels of O_2_ and CO_2_ within the fruit and these changes are usually accompanied by a net reduction of the O_2_ to CO_2_ ratio and by the accumulation of ethanol inside the fruit. The beneficial effects of low atmospheres of high O_2_ and CO_2_ inside the fruit lead to a reduction in the respiration rate, ethylene production and harmful effects, such as ethanol accumulation. Detecting ethanol in an internal fruit atmosphere may provide an additional measure of maturity.

While ethylene and ethanol regulates the fruit’s growth, ammonia is a common byproduct of the metabolism of the nitrogenous compounds and is involved (with proteins) in plant urea metabolism. The urea pathway has been studied not only in mammals and amphibians, but also in birds, invertebrates, insects, plants, yeast, fungi and even microorganisms. Compared to urea, ammonia is smaller, more volatile and more mobile [19].

Over a number of years, diverse techniques were utilized to identify volatile organic compounds created by living organisms under a variety of conditions. These methods for gas detection include different analytical tools [20], but techniques based on laser systems are used successfully in many directions of research [15,19,20].

Laser photoacoustic spectroscopy (LPAS) has the ability to monitor trace gases with high importance for biologic and medical applications. Because it is a sensitive and selective technique, it has limits ranging from the part-per-million (ppm, µmol/mol) to less than part-per-billion (ppb, nmol/mol) and is able to detect one or several gases and is used successfully increasing in fields [20,21]. The objective of this study is demonstrating the potential of laser-based photoacoustic spectroscopy in combination with multiphysics numerical simulation in detecting volatile organic compounds, such as ethylene, ammonia and ethanol emitted from Golden Apples during their maturation process.

## 2. Materials and Methods

In the present study, we performed analyses on “Golden Delicious”. The apples were taken from the supermarket with the specifications: Country of origin: Romania, Origin: Voineşti–Dâmboviţa.

The fruit are yellow in color, with rust spots; the pulp is yellowish, crispy with a pleasant aroma, medium strength and medium fruit size 133 g, spherical-conical shape. The “Golden Delicious” apples are preferred by most consumers.

Fruits were obtained from international producers (supermarkets). All apple fruit used in these measurements were stored at room temperature (25–27·°C) for subsequent use. The fruit were imported into a glass cuvette with a volume of 150 cm^3^ at room temperature and then connected to the LPAS system (a homemade system). This system is used for the sensitive detection of ethylene, ethanol and ammonia from the fruit to explore the role of these volatiles in fruit development.

CO_2_ laser photoacoustic spectroscopy has known a great increase over the years, in principal by enlarging the application fields gaining importance when it is applied to life sciences being able to detect a minimum concentration of 0.9 ppbV.

**Figure 1:**
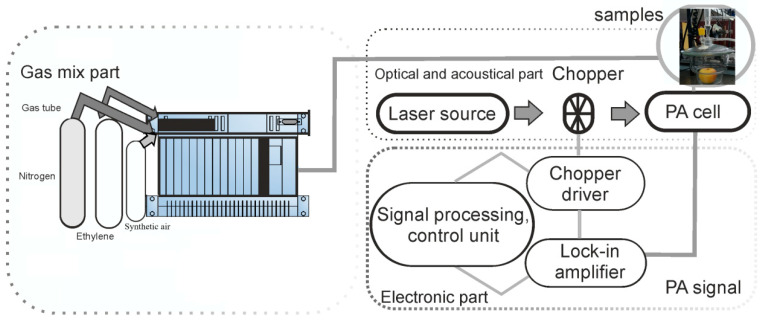
Block diagram of the CO_2_ laser photoacoustic system.

The continuous wave CO_2_ laser (LIR 25 SF, a homemade laser) line-tunable, frequency stabilized emitting radiation in the 9.2–10.8 µm region on 54 different vibrational–rotational lines with powers varying between 0.5 and 6.5 W [19,22]. The CO_2_ laser beam (a homemade laser) is modulated in intensity by a mechanical chopper operating at the appropriate resonant frequency of the cell (564 Hz), is focused by a ZnSe lens (homemade lens) and then introduced in the photoacoustic cell provided with four microphones where the acoustic wave is detected and to generate a corresponding electrical output signal. This signal is fed into a lock-in amplifier (Stanford Research Systems model SRB30 DSP, Sunnyvale, CA, USA) that gives the amplitude and the phase of the photoacoustic signal. A power meter measures the laser beam power after the photoacoustic cell. Its digital output is plugged in the data acquisition interface module along with the output from a lock-in amplifier. All experimental data are processed and stored by a computer (Figure 1).

Gas handling and vacuum systems are used to provide gas purity in the photoacoustic cell, to pump out the cell, set the sample gas in the photoacoustic cell at a controlled flow rate and monitor the pressures of gas mixtures. The gas handling system also includes two gas flow controllers (MKS Instruments Deutschland GmbH, MÜNCHEN, Germany) which limit the transfer of the sample in the cell to a flow rate of 36 L/h (600 sccm: standard cubic centimeters per minute) and the cuvette where the fruit is placed in order to be analyzed.

The CO_2_ photoacoustic spectroscopy system is characterized by the resonance frequency f = 564 Hz, quality factor of the system Q = 16.1, cell constant C = 5.41 × 10^3^ (Pa·cm/W) cell, microphones responsivity 4 × 20 × 10^−3^ = 8 × 10^−2^ (V/Pa), responsivity R = 433 cm·V/W, The pressure of the gas inside the cell influences the responsivity R of the photoacoustic cell. The laser beam interacts with the gas sent to the PA cell. The minimum detectable concentration of ethylene, ethanol or ammonia can be deduced as the ratio between minimum measurable voltage signal and the product of absorption coefficient, laser power and responsivity c_min_ = V_min_/α*RP_L_*, where *V* (V) is the photoacoustic signal, α (cm^−1^ atm^−1^)is the absorption coefficient at the laser wavelength, *c* (atm)represents the trace gas concentration (usually given in units of per cent, ppmV, ppbV or pptV), *P_L_* (W) is the unchopped laser beam power and *R* (V·cm/W) is the responsivity of the PA-photoacoustic cell [19,20,21,22,23].

**Figure 2:**
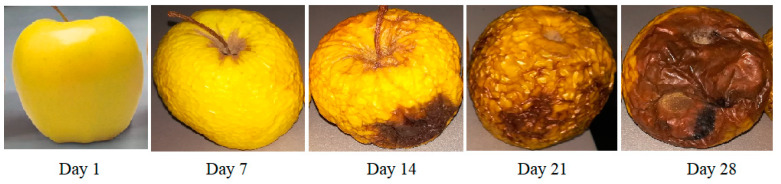
Pictures representing the evolution of a Golden Delicious apple over 35 days. The external appearance of apples that continues to degrade presenting speckles and wrinkles.

The CO_2_ laser photoacoustic spectroscopy system [19,20,22] was used to quantify the production of ethylene, ethanol and ammonia that are normally produced by apple fruit in normal aerobic conditions (synthetic air flow) over time (day 1, 7, 14, 21, 28 and 35, see Figure 2).

To evaluate the fruit tissue signal response from the glass cuvette (connected to the photoacoustic cavity (homemade cavity), we carefully removed all kinds of residual volatiles from the gas line connecting the gas flow system with the PA cell by flushing this line with pure nitrogen at atmospheric pressure for a few minutes. After the system was flushed, we transferred the gas from the glass cuvette to the PA cell with a flow of pure synthetic air.

Performing trace gas detection by photoacoustic spectroscopy requires precise knowledge of the absorption spectrum of each gas species to be determined. For a given concentration of that gas, the absorption spectrum is obtained by measuring the photoacoustic signal over all available laser source wavelengths. Measurements of absorption coefficients for all wavelengths of the laser source facilitate the determination of maximum absorption coefficient and permits to detect with high accuracy individual trace gas concentrations from a multicomponent mixture.

The assessment of absorption coefficients of ethylene, ethanol and, ammonia was performed for specific CO_2_ laser lines. The PA cell was filled at atmospheric pressure with certified gas mixtures of 0.996 ppmV ethylene in nitrogen and, 10.5 ppmV ethanol in nitrogen and, 10 ppm ammonia in nitrogen. Using the software user interface that allows recording the laser power, the PA signal and the calculated absorption coefficients on different panels we can determine the specific line where ethylene, ammonia and ethanol have their maximum absorption coefficients.

Transpiration is the process by which fresh fruit lose moisture, processes that include the transport of moisture through the fruit skin and evaporation to the surrounding air. Metabolic activity in fresh fruit continues for a short period after harvest. In the present work we simulate the chemical reaction between ethylene and water vapors using Chemical Reaction Engineering Module form Comsol 5.1 [24]. The main goal of this analysis is to validate the LPAS measurements accuracy by evaluation the reaction results. In the gas phase there is a reversible chemical reaction between ethylene, water vapor and ethanol (see Equation (1))

C_2_H_4_ + H_2_O <=> C_2_H_5_OH (1)

The chemical reaction was implemented using input parameters those used in the LPAS measurements, such as pressure, temperature, the cuvette volume. We solve the model using a time-dependent study step.

## 3. Results

### 3.1. Gas Detection Using CO_2_ Photoacoustic Spectroscopy

The physiological response of fruit under aerobic conditions was investigated using CO_2_ photoacoustic spectroscopy in order to evaluate the ethylene, ethanol and ammonia gas concentrations that were produced by golden apple fruit in the different stages of ripening.

The volatiles were investigated for six “Golden Delicious” apples over 35 days. Long-term experiments provide insight into the processes that produce changes in mature fruit and helped us to understand the significance of volatiles that differ in the rate of production under non-stressful conditions.

**Figure 3:**
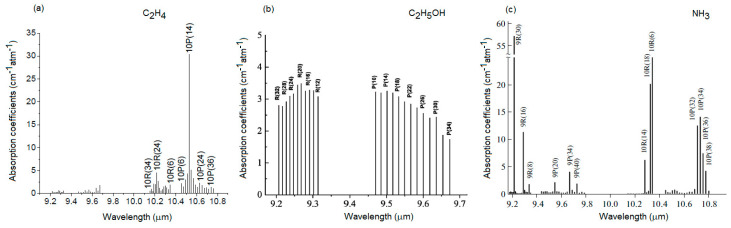
Absorption spectra of gases at CO_2_ laser wavelengths. (**a**) Absorption spectra of ethylene, (**b**) Absorption spectra of ethanol, (**c**) Absorption spectra of ammonia.

Ethylene, ethanol and ammonia emissions were established by introducing “Golden Delicious” apples into the cuvette sample and flushed with synthetic airflow at atmospheric pressure, and the resulting gas from the glass samples was transferred in the cell and analyzed. For each gas detection, the laser lines were chosen at the wavelengths where the gas to be analyzed has the maximum absorption coefficient (Figure 3).

The carbon dioxide laser operates in rotational-vibrational transition. Each vibrational transition consist of bands of discrete wavelengths resulting from a manifold of rotational structures, where lasing action can be obtained on more than a hundred lines. This laser operates in the middle infrared wavelength region with the principal wavelength bands centering around 9.4 and 10.6 µm. The CO_2_ laser transitions are 961 cm^−1^ transition of the 1.4 µm band and 1064 cm^−1^ transition of the 9.4 µm band. Because of the symmetry of the CO_2_ molecule, laser transitions occur to lower energy levels whose rotational numbers are even, resulting in more than 30 lines in each of the two branches P and R.

For ethylene detection laser line was determined at 949.479 cm^−1^, where we have the maximum absorption coefficient of 30.4 cm^−1^ atm^−1^ [19,20]. Ethanol was determined at 9R(22) laser line with a maximum absorption coefficient of α = 4.081 cm^−1^ atm^−1^ [20,23] and the maximum absorption coefficient of ammonia α = 57.1 cm^−1^ atm^−1^ was established at 9R(30) [22].

**Figure 4:**
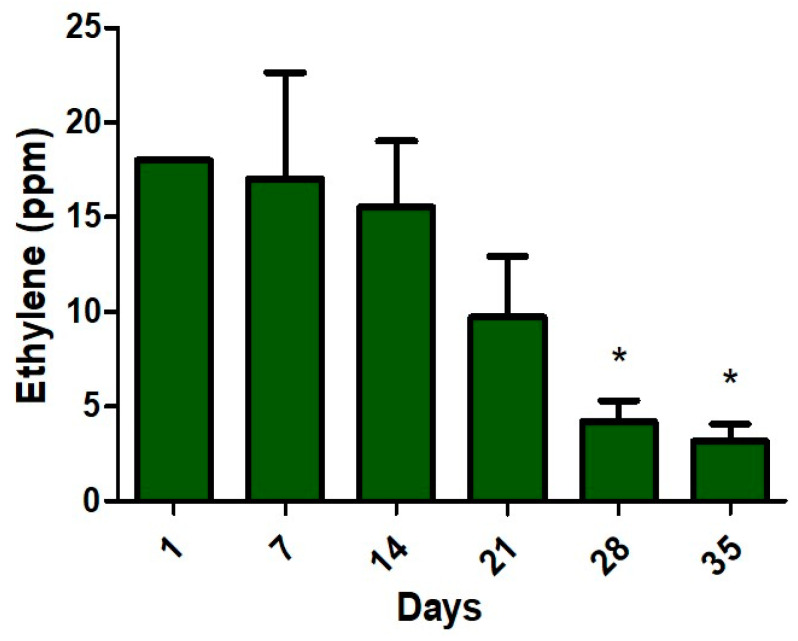
Ethylene concentrations emitted from “Golden Delicious” apples over 35 days under normal conditions. The error bar for day 1 is not visible due to a very small error. * *p* < 0.05 for unpaired *t-*test with Welch’s correction.

Figure 4 shows the level of ethylene gases emitted by all six “Golden Delicious” apple fruit ranging from an initial weight of about 133 g to 89 g at the end of the experiment.

All the data are presented as mean ± standard deviation if not stated otherwise, from at least 4 independent experiments. The statistical analysis was performed using GraphPad Prism 5 software (GraphPad Software, Inc., San Diego, CA, USA) and applying the unpaired t-test with Welch’s correction. *p* < 0.05 was considered statistically significant.

Starting with day 28 the ethylene concentration decreases, the apples become speckled and wrinkled.

During the experiment, a degradation of the apples can be observed, evidenced by the decrease of the concentration of ethylene, the staining and the wrinkling of the apple, but also by the weight loss of the apples.

The ethanol emission was measured at the same time with ethylene by changing the laser line. Ethanol molecules present a strong absorption 9R(22) laser line where the absorption coefficient is α = 4.081 cm^−1^ atm^−1^.

**Figure 5:**
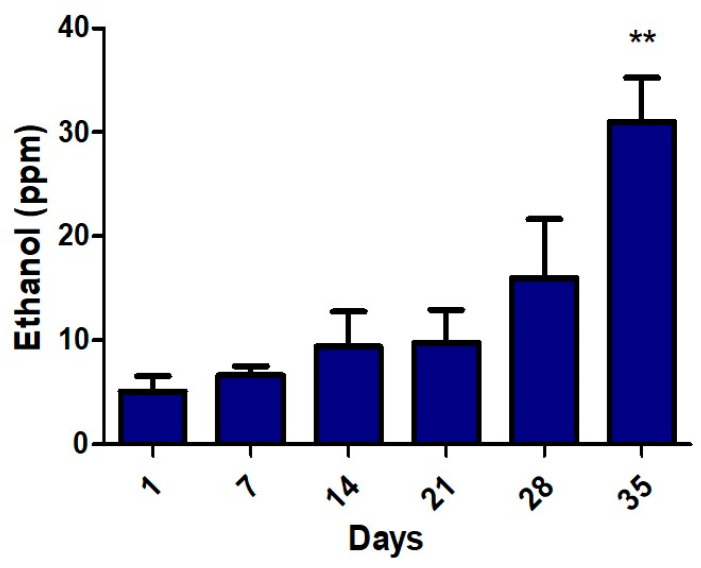
Ethanol concentrations emitted from “Golden Delicious” apples over 35 days under normal conditions. ** *p* < 0.01 for unpaired *t-*test with Welch’s correction.

In Figure 5 the release of ethanol molecules over 35 days was recorded and the process of degradation was studied.

In all cases the ethanol presents a constant increase in concentration providing an early indicator for fermentation. At the end of the experiment when the apple present spots and wrinkles a maximum in ethanol emission was observed.

The experiment reveals that the level of ethanol increases in day 35 when the ethylene concentration is minimal. The production of ethanol stops the process of fruit ripening.

During fruit maturation, the ethanol accumulates at low levels and plays an important role in the postharvest stage of apples. From our experiments, it can be observed that the ethanol emission is steadily increasing with apple degradation reaching a maximum value of 34 ppm on day 35.

The ammonia concentration from apples was measured at the same time with ethylene and ethanol by changing the laser line to 9R(30) laser line where the ammonia absorption coefficient is α = 57.1 cm^−1^ atm^−1^.

**Figure 6:**
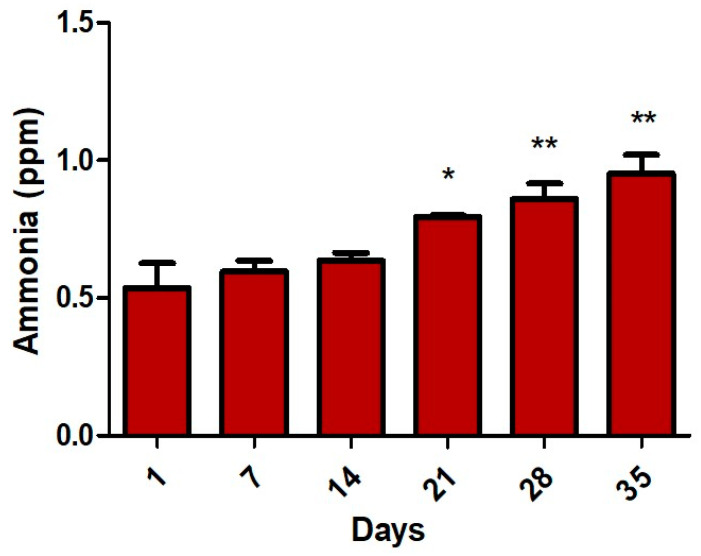
Ammonia concentrations emitted from “Golden Delicious” apples over 35 days under normal conditions. * *p* < 0.05, ** *p* < 0.01 for unpaired *t-*test with Welch’s correction.

Figure 6 shows the level of the ammonia gases emitted by apple fruit over 35 days under normal conditions.

It can be observed that the ammonia content in the internal atmosphere over 35 days slowly increases starting with day 21.

### 3.2. Numerical Simulation and Experimental Verification of Chemical Reactions

Using numerical simulations, we studied whether there is a possible chemical reaction between ethylene and water vapors found in apple respiration, a reaction that could influence the concentrations of ethylene and ethanol.

The numerical study was performed depending on time, the chosen interval being 1:35 days, interval identical to the one in which the LPAS measurements were realized. In addition, the numerical simulations were performed in the same temperature and pressure conditions, at room temperature and atmospheric pressure.

**Figure 7:**
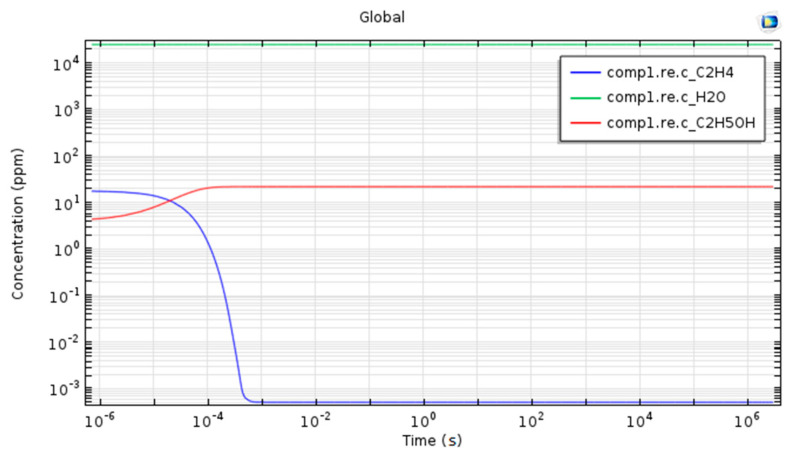
Time dependence of ethylene, ethanol and water vapor concentrations plotted on a logarithmic scale over a period of 35 days.

Figure 7 shows the time evolution of ethylene, ethanol and water vapors concentrations over a period of 35 days.

The results obtained from the numerical simulations show that there is no chemical reaction that influences the concentrations of ethylene and ethanol in apple respiration at room temperature and atmospheric pressure and the concentrations of these two gases are due to the respiration process in apples. In the conditions that were carried out in the experiments, all gases from the apple respiration behave independently.

## 4. Discussion

The current research describes an experimental study of the detection of ethylene, ethanol and ammonia released by Golden Delicious apples using laser photoacoustic spectroscopy. This technique is very precise allowing each gas to be measured with high efficiency. In a complex mixture of gases, a proper absorption laser line must be chosen for the determination of the individual concentration so that there are little or no interferences with other gases [24,25,26,27,28].

The detection of ethylene, ethanol and ammonia from apples presents interest for better understand internal fruit development in storage conditions. These volatiles have important roles in the maturation of apples. Ethylene is an important factor that produces changes in climacteric fruit, but ethanol and ammonia also have their influences.

Ethanol is capable of retarding senescence and inhibiting ethylene production in plants, leading to injury symptoms in fruit. Ammonia is a product of protein catabolism associated with the senescence of fruit and it accumulates during postharvest handling.

During 35-day trials, golden apple ethylene emissions showed a typical climacteric growth during the first seven days and then began to fall, while the ethanol production of apples increased gradually.

From day 21 on, apples creased and lost weight. Starting on day 28, the apples becomes speckled and wrinkled, at day 35 their weight had dropped to about 89 g.

From our measurements on ethylene phytohormone analysis, we can observe that ethylene emission is lower when the apples are more speckled and wrinkled. The ethanol normally accumulates at low levels during fruit maturation and plays an important role in the postharvest stage of apples. An important aspect of the study on the internal processes of fruit is the emission of ethanol that increases constantly with the degradation of the apple reaching a maximum value of 34 ppm on day 35. Ammonia pile-up inside the stored fruit slowly increases over the 35 days period causing fruit spoilage and skin blackening.

The principal finding from our investigation is that at day 28, when the ethylene emission drops significantly and when ethanol emission increases sharply, is the point when apple development becomes suppressed. The ethylene decreases from 18 ppm to 4.2 ppm (77%) and the ethanol increases from 5 ppm to 16 ppm (220%).

A considerable number of investigations were conducted on the specific properties of the aging and ripening of fruit [28,29,30,31,32,33,34,35]. Ethylene, ethanol and ammonia emission from apples influences the internal activity of fruit leading also to a modification of the outer layer.

At the same time, we determined through numerical simulations using Comsol Multiphysics the conversion of ethylene to ethanol during the respiration of apples for a period of time. The simulation results were compared with those obtained by photoacoustic spectroscopy.

From a practical point of view, information about ethylene biosynthesis, ethanol and ammonia action in the internal apple atmosphere reported in this study can be used to create effective tools capable of evaluating and predicting the evolution of the degradation process. New opportunities can be developed for researchers to promote new and improved ways for nondestructive quality evaluation of fruit using gas sensing techniques.

## 5. Conclusions

In summary, CO_2_ laser photoacoustic spectroscopy was performed to give information about the internal processes occurring in Golden Delicious apples stored over long periods of time. At the same time, the conversion of ethylene to ethanol during respiration of apples was determined through numerical simulations using Multiphysics and compared with data obtained by photoacoustic spectroscopy.

Detection of these gases (ethylene, ethanol and ammonia) from apples using CO_2_ laser photoacoustic spectroscopy hold promise for better understanding internal fruit development in storage conditions. These volatiles have important and different roles in apple processes. The main benefit of CO_2_ laser photoacoustic spectroscopy on the “Golden Delicious” apple’s internal atmosphere is that has the ability to determine the low concentration of ethylene, ethanol and ammonia. It was found that as the concentration of ethanol increased, ethylene production decreased after reaching a maximum of 23 ppm—and ammonia slowly increased. Assessment in the endogenous volatile compounds from the fruit may demonstrate that the quality and quantity of volatiles may be linked to postharvest management of fruit. New information about these volatiles will help us in achieving the best shelf life of fruit.

Very little is known about the influence of different volatiles in regulating the processes in fruit. Ethylene remains a major controlling factor in climacteric fruit processes, but its biosynthesis, perception, sensitivity are influenced by other endogenous volatiles. Finding as much information about these volatiles as possible will help us in achieving the best shelf life of fruit.

Developing fruit-specific and real-time systems to manipulate levels of important gases during storage will allow further improvements in shelf life and quality of stored fruit to be attained.

Overall, the results of this research show that it is possible to assess volatile molecules that can further our understanding of postharvest management processes of fruit.

## References

[1] Slavin J.L., Lloyd B. (2012). Health benefits of fruits and vegetables. Adv. Nutr..

[2] Arah I.K., Amaglo H., Kumah E.K., Ofori H. (2015). Preharvest and postharvest factors affecting the quality and shelf life of harvested tomatoes: A mini review. Int. J. Agron..

[3] Paul V., Pandey R. (2014). Role of internal atmosphere on fruit ripening and storability—a review. J. Food Sci. Technol..

[4] Biale J.B. (1964). Growth, maturation and senescence in fruits. Science.

[5] Abeles F.B., Morgan P.W., Saltveit M.E. (1992). Ethylene in Plant Biology.

[6] Lelievre J.M., Latche A., Jones B., Bouzayen M., Pech J.C. (1997). Ethylene and fruit ripening. Physiol. Plant..

[7] Prasanna V., Prabha T.N., Tharanathan R.N. (2007). Fruit ripening phenomena-An overview. Crit. Rev. Food Sci. Nutr..

[8] Paul V., Pandey R., Srivastava G.C. (2012). The fading distinctions between classical patterns of ripening in climacteric and non-climacteric fruit and the ubiquity of ethylene—An overview. J. Food Sci. Technol..

[9] El-Ramady H.R., Domokos-Szabolcsy É., Abdalla N.A., Taha H.S., Fári M. (2015). Postharvest management of fruits and vegetables storage. Sustain. Agric. Rev..

[10] Weber A., Brackmann A., Both V., Pavanello E.P., Anese R.O., Schorr M.R.W. (2016). Ethanol educes ripening of ‘Royal Gala’ apples stored in controlled atmosphere. Anais da Academia Brasileira de Ciências.

[11] Grant A. 2018 Gardening Know How. https://www.gardeningknowhow.com/edible/fruits/fegen/ethylene-gas-information.htm.

[12] Khan M.I.R., Trivellini A., Fatma M., Masood A., Francini A., Lqbal N., Ferrante A., Khan N.A. (2015). Role of ethylene in responses of plants to nitrogen availability. Front. Plant Sci..

[13] Iqbal N., Khan N.A., Nazar R., Silva J.A. (2011). Ethylene-stimulated photosynthesis results from increased nitrogen and sulfur assimilation in mustard types that differ in photosynthetic capacity. Environ. Exp. Bot..

[14] Iqbal N., Khan N.A., Ferrante A., Trivellini A., Francini A., Khan M.I.R. (2017). Ethylene role in plant growth, development and senescence: Interaction with other phytohormones. Front. Plant Sci..

[15] Popa C. (2019). Ethylene measurements from sweet fruits flowers using photoacoustic spectroscopy. Molecules.

[16] Dixon J., Hewett E.W. (2000). Factors affecting apple aroma/flavour volatile concentration: A Review. N. Z. J. Crop Hortic. Sci..

[17] Chang C. (2016). Q&A: How do plants respond to ethylene and what is its importance?. BMC Biol..

[18] Jackson M.B., Herman B., Goodenough A. (1982). An examination of the importance of ethanol in causing injury to flooded olants. Plant Cell Environ..

[19] Dumitras D.C., Dutu D.C., Matei C., Magureanu A.M., Petrus M., Popa C. (2007). Laser photoacoustic spectroscopy: Principles, instrumentation, and characterization. J. Optoelectron. Adv. Mater..

[20] Popa C., Bratu A.M., Petrus M., Bacalum M. (2020). The analysis of lead phytotoxicity in seeds using CO_2_ laser photoacoustic spectroscopy. Molecules.

[21] Dumitras D.C., Banita S., Bratu A.M., Cernat R., Dutu D.C.A., Matei C., Patachia M., Petrus M., Popa C. (2010). Ultrasensitive CO_2_ laser photoacoustic system. Infrared Physics Technol. J..

[22] Dumitras D.C., Dutu D.C., Matei C., Cernat R., Banita S., Patachia M., Bratu A.M., Petrus M., Popa C. (2011). Evaluation of ammonia absorption coefficients by photoacoustic spectroscopy for detection of ammonia levels in human breath. Laser Phys..

[23] Ivascu I.R., Matei C.E., Patachia M., Bratu A.M., Dumitras D.C. (2016). CO_2_ laser photoacoustic measurements of ethanol absorption coefficients within infrared region of 9.2–10.8 μm. Spectrochim. Acta Part A Mol. Biomol. Spectrosc..

[24] Nur S.H., Ayub M.S., Zalizawati A. Ethanol production via direct hydration of ethylene: A review. Proceedings of the International Conference on Global Sustainability and Chemical Engineering (ICGSE).

[25] Gordon I.E., Rothman L.S., Hill C., Kochanov R.V., Tan Y., Bernath P.F., Birk M., Boudon V., Campargue A., Chance K.V. (2017). The HITRAN2016 molecular spectroscopic database. J. Quant. Spectrosc. Radiat. Transf..

[26] Legé K.E., Cothren J.T., Morgan P.W. (1997). Nitrogen fertility and leaf age effect on ethylene production of cotton in a controlled environment. Plant. Growth Regul..

[27] Lea U.S., Slimestad R., Smedvig P., Lillo C. (2007). Nitrogen deficiency enhances expression of specific MYB and bHLH transcription factors and accumulation of end products in the flavonoid pathway. Planta.

[28] Cvikrová M., Binarová P., Eder J., Vágner M., Hrubcová M., Zo J. (1997). Effect of inhibition of phenylalanine ammonia-lyase activity on growth of alfalfa cell suspension culture: Alterations in mitotic index, ethylene production, and contents of phenolics, cytokinins and polyamines. Physiol. Plant..

[29] Davies F.T., He C., Chau A., Heinz K.M., Cartmill A.D. (2004). Fertility affects susceptibility of chrysanthemum to cotton aphids: Influence on plant growth, photosynthesis, ethylene evolution, and herbivore abundance. J. Am. Soc. Hortic. Sci..

[30] Bruinsma J., Lieberman M. (1983). Hormonal Regulation of senescence, ageing, fading, and ripening. Post-Harvest Physiology and Crop Preservation. Nato Advanced Study Institutes Series (Series A: Life Sciences).

[31] Mikal E., Saltveit J. (1989). Effect of alcohols and their interaction with ethylene on the ripening of epidermal pericarp discs of tomato fruit. Plant Physiol..

[32] Pesis E., Marinansky R. (1993). Inhibition of tomato ripening by acetaldehyde vapour or anaerobic conditions prior to storage. J. Plant Physiol..

[33] Podd L.A., Van Staden J. (1998). The role of ethanol and acetaldehyde in flower senescence and fruit ripening—A review. Plant Growth Regul..

[34] Alexander L., Grierson D. (2002). Ethylene biosynthesis and action in tomato: A model for climacteric fruit ripening. J. Exp. Bot..

[35] Karaoulanis G.D., Dilley D. (1993). Ethanol content of ripening apples (variety Mutsu) and ethylene production during storage under anaerobic conditions at room temperature. Int. J. Refrig..

